# Microbiological characteristics of sepsis in a University hospital

**DOI:** 10.1186/s12879-015-0798-y

**Published:** 2015-02-14

**Authors:** Adriana Valderez Reis Vendemiato, Angela von Nowakonski, Fernando Augusto de Lima Marson, Carlos Emilio Levy

**Affiliations:** Methodist University of Piracicaba, Piracicaba, SP Brazil; Clinical Microbiology Laboratory, Clinical Hospital, State University of Campinas, Campinas, SP Brazil; Department of Medical Genetics and Department of Pediatrics, Faculty of Medical Sciences – State University of Campinas, Campinas, SP Brazil; Department of Clinical Pathology, Faculty of Medical Sciences, State University of Campinas, Tessália Vieira de Camargo 126, “Zeferino Vaz”, Barão Geraldo, Campinas, São Paulo CEP 13083-887 Brazil

**Keywords:** Sepsis, Antimicrobial resistance, Blood cultures, Etiological agent

## Abstract

**Background:**

Microbiological characteristics of sepsis and antimicrobial resistance are well studied, although in State University of Campinas, no data has been published yet.

**Methods:**

The main agents related to sepsis and antimicrobial resistance were analyzed. The blood culture records requested from 4,793 hospitalized patients were analyzed. The samples were processed using the Bact/Alert® system for agent identification and antimicrobial susceptibility.

**Results:**

A total of 1,017 patients met the inclusion criteria for a sepsis diagnosis, with 2,309 samples tested (2.27 samples/patient). There were 489 positive samples (21% positive) isolated from 337 patients (33.13%), but more rigorous criteria excluding potential contaminants resulted in analysis being restricted to 266 patients (315 agents). The prevalent microorganisms were coagulase negative *Staphylococcus* (CNS) (15.87%), *Escherichia coli* (13.0%), *Staphylococcus aureus* (11.7%), *Klebsiella pneumoniae* (9.8%), *Enterobacter sp* (9.5%), *Acinetobacter baumannii* (9.2%), *Pseudomonas aeruginosa* (5.7%) and *Candida sp* (5.1%). Examining antimicrobial resistance in the agents revealed that 51% of the *S. aureus* isolates were methicillin-resistant *S. aureus* (MRSA) and 80% of the CNS isolates were oxacillin-resistant. For *A. baumannii*, the ideal profile drugs were ampicillin sulbactam and piperacillin/tazobactam, and for *P. aeruginosa*, they were piperacillin/tazobactam and ceftazidime. Enterobacteria showed on average 32.5% and 35.7% resistance to beta-lactams and ciprofloxacin, respectively. When all Gram-negative bacteria were considered, the resistance to beta-lactams rose to 40.5%, and the resistance to ciprofloxacin rose to 42.3%.

**Conclusions:**

Eighty percent of the agents identified in blood cultures from patients with sepsis belonged to a group of eight different agents*.* For empirical treatment, carbapenems and vancomycin unfortunately still remain the best therapeutic choice, except for *A. baumannii* and *P. aeruginosa*, for which piperacillin/tazobactan is the best option.

## Background

Sepsis is one of the most serious and urgent infectious events in clinical practice, and its diagnosis is traditionally based on blood culture, defined as the presence (probable or documented) of infection together with systemic manifestations of infection [1]. Efforts for the early recognition of sepsis using biomarkers (IL6, high-sensitivity CRP, PCT) as a molecular method for detection as well as identifying etiologic agents and resistance markers are important approaches for the diagnosis of this syndrome [2-12]. A simple bacteremia, which may originate from the digestive tract and progress to a state of septic shock and multiple organ dysfunction, involves important variables, such as the agent involved, quantity and virulence, type of invasion (continued or intermittent), available diagnostic resources, host response to infection and the precocity and adequacy of clinical-therapeutic interventions. These are some of the determining factors influencing clinical outcome [9]. Therefore, blood culture is a laboratory technique of great importance and is one of the most frequently ordered tests in the clinical microbiology laboratory [10]. Similarly, microscopic examination following Gram staining on positive blood cultures can quickly guide the clinician in choosing an empirical antimicrobial therapy [13]. Recently, other laboratory approaches involving molecular techniques have been incorporated, but these procedures remain limited and costly [9,10,13,14]. These molecular techniques may have a significant impact by increasing the early diagnosis and detection of etiologic mechanisms of resistance. Some important factors in the detection of blood cultures include the number of samples, volume, collection site, use of automated systems for monitoring and type of incubation atmosphere (anaerobic or not). Other factors may interfere to give false positive or negative results, such as the use of antibiotics and antisepsis failures, and laboratory limitations may delay diagnosis or give incorrect information [15-18].

There are few data on the annual incidence of bloodstream infections in the world, but sepsis is a leading cause of death in hospitalized patients. In the U.S., approximately 750,000 cases of bloodstream infections occur each year, resulting in 215,000 deaths [9,14]. In Brazil, the current data show an incidence density of sepsis from 57.9 per 1,000 patient-days, and the mortality rate of patients with systemic inflammatory response syndrome (SIRS), sepsis, severe sepsis and septic shock was 24.2%, 33.9%, 46.9% and 52.2%, respectively [19]. Brazilian and international studies generally emphasize the clinical, epidemiological and diagnostic resources, with limited analyses of both the agents involved and the antimicrobial resistance of the agents; such analyses usually involve non-sequential samples [19-24]. In this context, our objective was to analyze the main agents related to sepsis and the antimicrobial resistance profile of those agents in a public university hospital.

## Methods

Blood culture records that were requested from hospitalized patients in the Intensive Care Units (ICUs), Outpatient Emergency Unit (OEU) and the wards of the University Hospital during the course of one year were analyzed retrospectively. The blood culture samples were processed by the Microbiology Laboratory using the Bact/Alert® (bioMérieux, USA) automated detection equipment. Samples detected by the equipment were immediately subcultured on blood agar, chocolate agar and MacConkey agar plates for bacterial and fungal isolation, and the bacterioscopy microscopic examination results were reported immediately as preliminary results to the doctor. The isolated colonies, 12 to 24 hours incubation after incubation, were identified and evaluated for antimicrobial susceptibility using Vitek® II (bioMérieux, USA) or by the disk diffusion test according to the standards of the Clinical and Laboratory Standards Institute (CLSI), which are updated annually.

The blood culture data were compared with information obtained from the original processing worksheets from the Laboratory of Microbiology and the Bact/Alert® (bioMérieux, USA) database. Exact Fisher Test was used to compare positive sample among medical specialties. These data were analyzed using the SPSS (Statistical Package for Social Sciences) v.21.0 (IBM Corp. Released 2012. IBM SPSS Statistics for Windows, Version 21.0. Armonk, NY, USA). StatCalc – EpiInfo 7 (Atlanta, GA, USA) calculated the power of sample. For the 4,793 enrolled, the positive sepsis diagnosis for 1,017 (21.21%) has a confidence interval of 99.99%.

The inclusion criterion was a diagnosis of sepsis, excluding all other diagnoses such as bacteremia, fever and repeated samples for the same patient within one month, except in cases of agent change. In our casuistic was not possible to characterize the septic shock and severe sepsis. In our study, the sepsis diagnosis was defined as the presence (probable or documented) of infection together with systemic manifestations of infection [1]. Cases with potential contamination, when isolated from a single sample, were excluded from the analysis. We considered the following potential contaminating agents: *Bacillus sp, Bacillus cereus, Corynebacterium sp, Micrococcus sp, Propionibacterium sp, Streptococcus viridans* and coagulase-negative *Staphylococcus* (CNS). Regardless of the number of positive samples for the same patient, only one strain per patient was considered for the analysis of the antimicrobial profile.

The study was authorized by the Ethical Committee from State University of Campinas without the inclusion of an informed consent.

## Results

A total of 16,046 blood culture samples were collected from 4,793 patients and processed by the Laboratory of Microbiology. A total of 1,017 patients met the inclusion criteria for the diagnosis of sepsis and included 2,309 samples (and an average of 2.27 samples collected per patient); 489 of these samples were positive (21% positive).

The medical specialties with the highest number of patients diagnosed with sepsis were OEU with 440 patients (43.26%), adult ICU with 134 patients (13.18%) and Neonatology with 104 patients (10.23%).

The average number of samples collected per patient ranged from 1.8 to 2.94. The specialties with the highest number of samples collected per patient were Neonatology and the adult ICU, with approximately three samples per patient. The specialties with the lowest average sample per patient were Pediatrics and OEU, with approximately 1.9 samples per patient.

The percentage of positivity was higher in the coronary unit with 50% positive samples, followed by the adult ICU with 30.93%. The lowest percentage of positive samples occurred in Internal Medicine and OEU (Table 1).

**Table 1:** **Distribution by specialties, number of patients, samples collected, positive samples, average per patient and percentage of positive blood cultures of 2,309 samples collected from 1,017 patients diagnosed with sepsis**

**Medical specialty**	**Patients**		**Samples collected**		**Positive samples**		**Positive average per patient**	**% of positivity**	p-value*	**OR (95% CI)**
	**N**	**%**	**N**	**%**	**N**	**%**				
OEU – UER	440	43.26	862	37.33	139	28.43	1.96	16.13	**<0.001**	**0.603 (0.481 – 0.753)**
Internal medicine	113	11.11	296	12.82	35	7.16	2.62	11.82	**<0.001**	**0.461 (0.309 – 0.669)**
Neonatology	104	10.23	306	13.25	82	16.77	2.94	26.8	0.0139	1.435 (1.075 – 1.903)
ICU adult	134	13.18	362	15.68	112	22.90	2.70	30.93	**<0.001**	**1.865 (1.439 – 2.409)**
ICU pediatric	34	3.34	73	3.16	16	3.27	2.15	21.92	0.969	1.046 (0.555 – 1.868)
Trauma surgery	31	3.05	67	2.90	18	3.68	2.16	26.87	0.315	1.381 (0.745 – 2.44)
Coronary unit	27	2.65	58	2.51	29	5.93	2.15	50.00	**<0.001**	**3.89 (2.219 – 6.821)**
Pediatric ward	16	1.57	29	1.26	5	1.02	1.81	17.24	0.801	0.773 (0.261 – 1.937)
Other	118	11.60	256	11.09	53	10.84	2.13	20.35	0.917	0.968 (0.689 – 1.343)
**Total**	1,017	100.0	2,309	100.0	489	100.0	2.27	21.18		

N = number of patients. *Statistical analysis performed by Exact Fisher Test. Positive association is shown in bold.

The 489 positive samples were isolated from 337 of 1,017 patients diagnosed with sepsis (33.13%), which left 680 patients with negative samples (66.87%). To analyze the cases with the most evidence of sepsis, we excluded 71 patients who had only one positive sample for potential contaminants, leaving 266/1,017 patients (26.15%) with potential pathogens. Of these 266 patients, 315 different agents were isolated in 418 positive samples (1.57 positive samples per patient). A total of 219 patients (82.33%) had only a single agent, 45 patients (16.91%) had two different agents and two patients (0.75%) had three different agents (Tables 2 and 3).

**Table 2:** **Distribution of the main pathogens detected in blood cultures from 266 patients diagnosed with sepsis**

**Agent**	**Number of patients**	**Percentage**
Coagulase negative *Staphylococcus*	50	15.87%
*Escherichia coli*	41	13.02%
*Staphylococcus aureus*	37	11.75%
*Klebsiella pneumoniae*	31	9.84%
*Enterobacter sp*	30	9.52%
*Acinetobacter baumannii*	29	9.21%
*Pseudomonas aeruginosa*	18	5.71%
*Candida sp*	16	5.08%
*Enterococcus sp*	14	4.44%
*Proteus-Providencia-Morganella*	12	3.81%
*Serratia marcescens*	8	2.54%
*Streptococcus pneumoniae*	8	2.54%
*Streptococcus viridans*	4	1.27%
*Stenotrophomonas maltophilia*	3	0.95%
*Streptococcus bovis*	3	0.95%
Other pathogens*	11	3.52%
**Total**	315	100.00%

*1 (0.32%) patient for the follow bacteria*: Aeromonas caviae, Burkhloderia cepacia, Citrobacter freundii, Corynebacterium sp, Cryptococcus neoformans, Fusarium sp, Haemophilus influenza, Listeria monocytogenes, Pseudomonas putida, Salmonella sp, Streptococcus pyogenes.*

**Table 3:** **Associations of bacteria isolated from 47 patients diagnosed with sepsis**

**Bacteria**	***S. marcescens***	***S. aureus***	***A. baumannii***	**SCN**	***E. faecalis***	***P. mirabilis***	***E. cloacae***	***E. coli***	***E. aerogenes***	**Total**
*E.coli*	1	-	-	-	-	-	-	-	-	1
*E. cloacae*	-	2	3	1	-	-	-	-	-	6
*K. pneumonia*	-	1	1	1	3	1	1	1	1	10
*A. baumannii*	-	-	-	3	-	-	-	-	2	5
*S. aureus*	-	-	-	2	-	1	-	-	1	4
*E. faecalis*	-	-	1*	4	-	-	-	-	-	5
*P. aeruginosa*	-	-	-	3	1**	-	-	-	1	5
*C. albicans*	-	-	-	1	-	1	-	-	-	2
*C. tropicalis*	-	-	1	1	-	-	-	-	-	2
*Proteus spp*	-	-	-	2	-	-	-	-	-	2
*S. pneumoniae*	-	-	1	-	-	-	-	-	-	1
*B. cepacia*	-	-	1	-	-	-	-	-	-	1
*Salmonella sp*	-	-	-	1	-	-	-	-	-	1
*A. caviae*	-	-	-	-	-	-	-	1	-	1
*P. putida*	-	-	-	-	-	-	1	-	-	1
**Total**	1	3	8	19	4	3	2	2	5	47

*Associated with *S. maltophilia.*

**Associated with *E. aerogenes.*

In the Pediatric areas (Neonatology, Pediatric ward and Pediatric ICU), 64 patients with positive samples were detected, but 50% of these patients were positive in only a single sample, which contained a potential Gram positive contaminant. Of the remaining 32 patients with positive samples, 19 had Gram positive isolates (*Staphylococcus aureus* - nine patients, CNS - eight patients (more than one positive sample of the same species) and *Enterococcus* - two patients), eight had Gram negative isolates (*Enterobacter spp* - four patients, *Enterobacter spp* + *Klebsiella pneumoniae* - one patient, *Pseudomonas aeruginosa* - one patient, *Escherichia coli* - one patient and *Haemophilus influenzae* - one patient), one patient had *S. aureus* and *Enterobacter spp*, three patients had *Candida albicans* and one patient had *C. albicans* and *Proteus mirabilis.*

The percentage of antimicrobial resistance in bacteria isolated from 221 patients diagnosed with sepsis is shown in Table 4.

**Table 4:** **Percentage of antimicrobial resistance in bacteria isolated from 221 patients diagnosed with sepsis**

**Bacteria**	**N**	**AMI**	**CPM**	**CAZ**	**PPT**	**CIP**	**IPM**	**MER**	**OXA**	**ASB**	**VAN**	**ESBL+**
*E. coli*	41	0	12	12	12	32	0	0	-	46	-	12
*K. pneumoniae*	31	3	70	70	70	56	0	0	-	77	-	71
*E. cloacae*	19	21	21	21	21	29	5	5	-	100	-	-
*E. aerogenes*	11	82	77	77	77	66	0	0	-	100	-	-
Other *enterobacteria*	21	5	5	5	5	5	0	0	-	43	-	-
*S. aureus*	37	-	-	-	-	-	-	-	51	-	0	-
*S* coag neg	40	-	-	-	-	-	-	-	80	-	0	-
*E. faecalis*	12	-	-	-	-	-	-	-	-	-	0	-
*E. faecium*	2	-	-	-	-	-	-	-	-	-	50	-
*A. baumannii*	29	52	80	80	30	77	69	65	-	23		-
*P. aeruginosa*	18	28	29	17	12	35	17	17	-	-		-

N = number of patients, AMI = amikacin, CPM = cefepime, CAZ = ceftazidime, PPT = piperacillin-tazobactam, CIP = ciprofloxacin, IPM = imipenem, MER = meropenem, OXA = oxacillin, ASB = ampicillin-sulbactam, VAN = vancomycin, ESBL = extended spectrum beta-lactamase.

## Discussion

Among the patients diagnosed with sepsis, 2/3 were from the OEU plus ICU adults and Neonatology, which are the most critical areas of the hospital. The difference in the average of two blood culture samples per patient in OEU and three blood culture samples in the adult ICU and Neonatology can be explained by the established standard of using two samples per event per patient in acute cases and three samples per event per patient for patients using a catheter and more seriously ill patients.

The percentage of positive samples was higher in the coronary unit (50%), probably because the sepsis diagnosis is less frequent, doctors are more confident about the diagnosis and the blood cultures are used more judiciously. The adult ICU had the next highest percentage of positive samples (31%). The lowest percentages occurred in Internal Medicine and OEU, with the latter being the area that accounted for the largest number of samples collected and had a less systematized attendance with overcrowding and overloaded conditions of service. In general, the positivity rate of blood cultures can vary greatly depending on the type and complexity of the institution and are, on average, 10 to 15%. Obviously, our rate exceeding 20% can be explained by the selection of cases with a diagnosis of sepsis, which generally does not exceed 50%, even in cases of septic shock [10].

Some microorganisms are more often associated with contamination (<5% chance of true bacteremia), including *Corynebacterium spp., Micrococcus spp., Bacillus spp.* and *Propionibacterium acnes,* especially when isolated in only one of two or more samples and when the sample becomes positive over 72 hours of incubation [12,15,17]. The *S. viridans, Enterococcus spp*. and CNS organisms account, on average, for 38%, 78% and 15% of true bacteremia [12], although these values may vary according to different authors and study populations [14-18]. Moreover, some microorganisms have a high positive predictive value for true bacteremia (>90%), even when they are isolated in only one sample. These organisms include *S. aureus, E. coli, Klebsiella spp* and other enterobacteria, *Neisseria meningitidis, Streptococcus pneumoniae, P. aeruginosa, Streptococcus pyogenes, Streptococcus agalactiae, Listeria monocytogenes*, *Neisseria gonorrhoeae, H. influenzae, Brucella spp, Candida spp* and *Cryptococcus spp* [12].

We discarded 7% of patients from our analysis who were considered to have blood cultures with potential contaminants by the criteria described above. However, this left 16% of the total cases with a CNS isolation, which still was the most frequently isolated agent in this series. This number of contaminants can be considered reasonable considering that over 40% of all samples taken were from OEU, where the collection conditions are less favorable [18].

The distribution by agents refers to a general hospital, including critical, immunosuppressed and transplant patients. Other factors can vary the distribution of agents, such as a higher number of cases of surgical infections (surgical hospitals) or community-acquired infections (pneumonia, meningitis and infections of skin and soft tissue), whereas the high number of infections by CNS may be associated with the use of catheters, especially those of long duration. The increasing isolation of non-fermenting bacteria (*P. aeruginosa* and *Acinetobacter baumannii* in approximately 15% of the total isolates) shows the presence of opportunistic agents associated with nosocomial infection and invasive procedures. In addition, opportunistic fungi of the genus *Candida* accounted for 5% of sepsis cases; 60% (9/15) were *C. albicans*, and 40% (6/15) were *Candida tropicalis*, which indicates the importance of these species in hospital isolates. The pediatric sampling is small and involves different sectors, but it shows a predominance of Gram positive bacterial infections that probably come from the community, whereas adult samples predominantly contain Gram negative bacteria, indicating a probably greater role of hospital infections.

Regarding the antimicrobial resistance results, we find that 51% of *S. aureus* are MRSA/ORSA (Methicillin-resistant *Staphylococcus aureus*/oxacillin-resistant *Staphylococcus aureus*), and we find an 80% methicillin/oxacillin resistance rate for CNS, which suggests it is worthwhile introducing an alternative drug (vancomycin) until the results of the antibiogram are available. The empirical therapy for *S. aureus* should consider the prevalence of MRSA strains in septic patients, which must be known on each unit or ward. The potentially most useful drugs for the treatment of major Gram negatives were carbapenems, with the exception of *A. baumannii*, which has better profile drugs such as ampicillin sulbactam and piperacillin/tazobactam. Enterobacteria showed, on average, 32.5% resistance to beta-lactams, and when all Gram negatives were considered, this resistance rose to 40.5%. The findings were similar for ciprofloxacin, with 35.7% of Enterobacteriaceae and 42.3% of all Gram negatives being resistant. Therefore, both third- and fourth-generation cefalosporins and quinolones are not recommended as the drug of choice for empiric therapy. The rare profiles of resistance were a vancomycin-resistant enterococci strain that, although endemic in our country, rarely causes sepsis and one case of *Enterobacter cloacae* that is resistant to carbapenems. This carbapenem-resistant *E. cloacoe* isolate was Hodge test negative and PCR negative for Kpc, which suggests that it does not produce a carbapenase. The mechanism of resistance is probably through the production of an AMPc-inducible beta-lactamase and the reduction of porin channels [25].

The recent literature contains little data on bacterial resistance in Brazilian hospitals or even in American or European hospitals, with most publications linked to pharmaceutical industry studies for the purpose of comparative drug performance research; these studies include other limitations, such as restrictions on etiologic agents, the healthcare of centers involved, non-sequential samples [19-24]. It is worth noting the difficulty of the empirical use of antimicrobials, considering the high percentage of strains of MRSA (51%) and about 40% of Gram-negative resistance to beta-lactam and quinolones drugs, requirement for the rapid transmission of information from microbiology laboratories such as the results of the Gram stain, the antibiogram, determining the minimum inhibitory concentration for rapid tests such as the Etest®, the testing for resistance mechanisms such as AMPc, ESBL, metallo-beta-lactamase, Kpc, VRE, MRSA, and fast and confident automation resources for blood cultures and bacterial identification are all critical for patient care, especially for cases of sepsis. The availability of experienced and attentive microbiologists to analyze and make decisions about unusual patterns of antimicrobial susceptibility results and analysis of unreliable automation results is essential, with special attention given to non-fermenting bacteria. This study was restricted to the microbiology ordering sheets analysis, the medical records were not examined and the patients were not clinically assessed. Therefore, it is not possible to properly classify their gravity, risk factors, therapeutic choices and outcomes. There were also limitations in the diagnosis of sepsis, but there are no markers at present for the accurate and early detection of sepsis, which is diagnosed based on clinical signs and laboratory data that are inadequately specific [9]. Cases of bacteremia may have been included, but the sample is representative of a continuous series of cases within one year of microbiological data follow-up at a tertiary general hospital. The accuracy of our routine diagnosis based on bioMérieux VITEK II and current literature [26] can be considered appropriate and in accordance with international standards, although more sophisticated techniques, such as mass spectrometry and amplification of nucleic acids, have recently been introduced; however, the impact of these new tests has yet to be assessed.

## Conclusions

Eighty percent of the agents identified in blood cultures from patients with sepsis belonged to a group of eight different agents*.* For empirical treatment, carbapenems and vancomycin unfortunately still remain the best therapeutic choice, except for *A. baumannii* and *P. aeruginosa*, for which piperacillin/tazobactan is the best option.

## Abbreviations

CLSI: Clinical and Laboratory Standards Institute
CNS: Coagulase negative *Staphylococcus*
CRP: C-reactive protein
ESBL: Extended-spectrum β lactamase
ICUS: Intensive care units
IL6: Interleukin 6
MRSA: Methicillin-resistant *Staphylococcus aureus*
OEU: Outpatient Emergency Unit
ORSA: Oxacillin-resistant *Staphylococcus aureus*
PCT: Procalcitonin
SIRS: Systemic inflammatory response syndrome
SPSS: Statistical package for social sciences
US: United States
USA: United States of American
VRE: Vancomycin-resistant enterococcus
